# Xanthogranulomatous prostatitis with multilocular mass on MRI

**DOI:** 10.1002/iju5.12791

**Published:** 2024-09-26

**Authors:** Takashi Okamoto, Kazuki Doi, Soma Ogura, Masafumi Kusunose, Kosuke Takahashi, Takuya Fujimoto, Mototsugu Muramaki, Yuji Yamada

**Affiliations:** ^1^ Department of Urology Hyogo Prefectural Amagasaki General Medical Center Amagasaki‐shi Hyogo Japan

**Keywords:** granulomatous, prostatic abscess, prostatitis, xanthogranulomatous, xanthoma

## Abstract

**Introduction:**

Xanthogranulomatous prostatitis is a very rare benign inflammatory lesion of the prostate that may be similar to prostatic carcinoma in clinical presentation and radiological characteristics.

**Case presentation:**

A 77‐year‐old man was admitted to our hospital because of high prostate‐specific antigen level. Magnetic resonance imaging showed a 6.5 cm in diameter multifocal mass with hemorrhage at the base of the left lobe of the prostate. Biopsy was performed. Histopathological examination revealed no evidence of malignancy. After biopsy, he developed recurring fever, so the patient was treated with open suprapubic tumor resection to control infection. Pathological examination revealed xanthogranulomatous prostatitis.

**Conclusion:**

It is necessary to diagnose xanthogranulomatous prostatitis by cooperation between urologists and pathologists, and consider xanthogranulomatous prostatitis as a differential diagnosis. Treatment should be conservative in principle; however, surgical intervention may be necessary.

**Keynote message**

Xanthogranulomatous prostatitis is a very rare benign inflammatory lesion of the prostate that can be similar to prostatic carcinoma in clinical presentation and radiological characteristics. We should be aware of xanthogranulomatous prostatitis as a differential diagnosis when malignant findings are not found on biopsy.

**Abbreviations & Acronyms**
ADC: appearant diffusion co‐efficient
COR: coronal
CT: computed tomography
DWI: diffusion‐weighted imaging
MRI: magnetic resonance imaging
SAG: sagittal
TRA: transverse
XP: xanthogranulomatous prostatitis

## Introduction

XP is a very rare benign inflammatory lesion of the prostate that can be similar to prostatic carcinoma in clinical presentation and radiological characteristics. Although XP has been reported, no study has reported a multilocular mass image on imaging.

## Case presentation

**Fig. 1:**
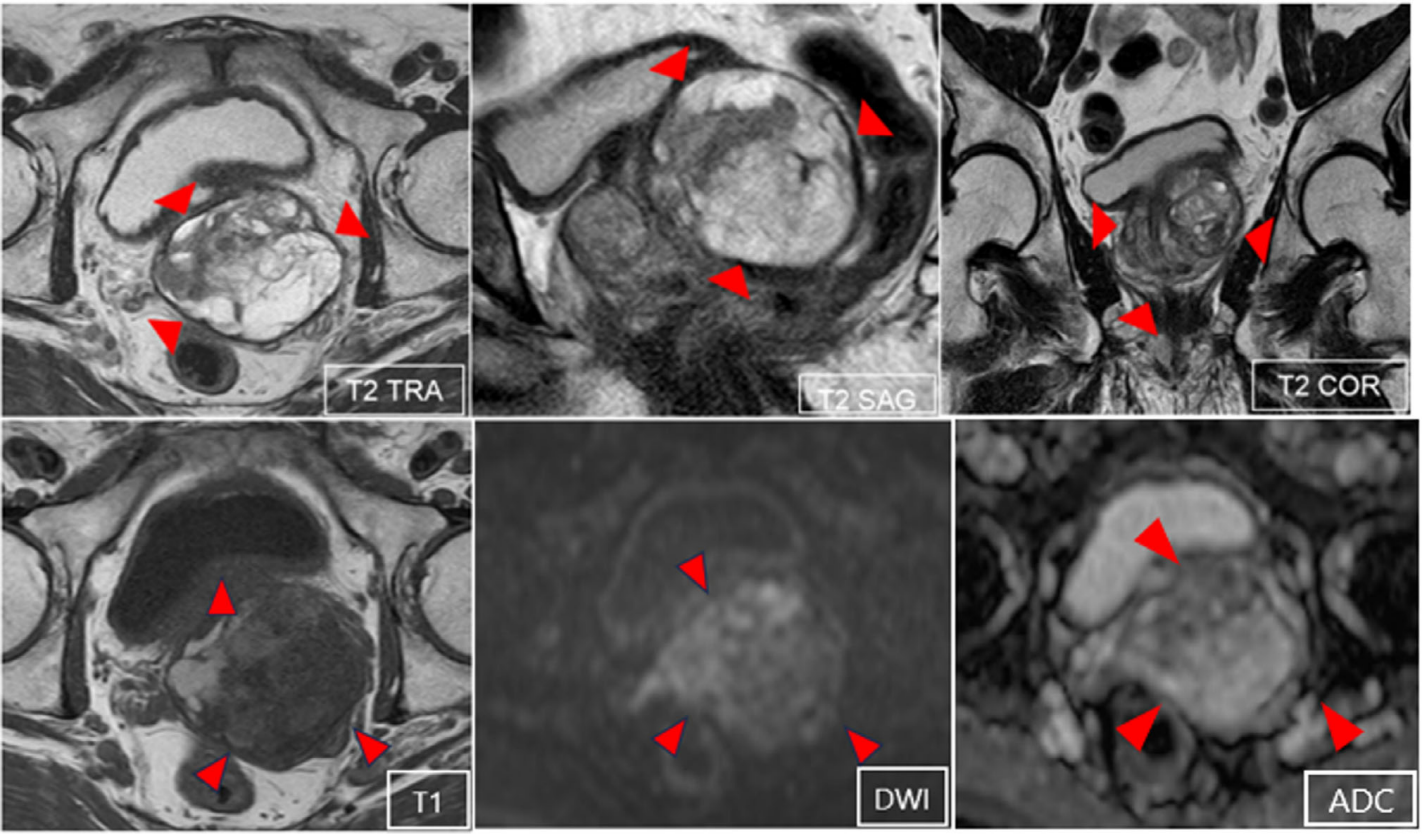
MRI revealed a 6.5 cm large multilocular mass with hemorrhage at the base of the left lobe of the prostate. ADC, Appearant diffusion co‐efficient; COR, coronal; DWI, Diffusion‐weighted imaging; RA, transverse; SAG, sagittal.

**Fig. 2:**
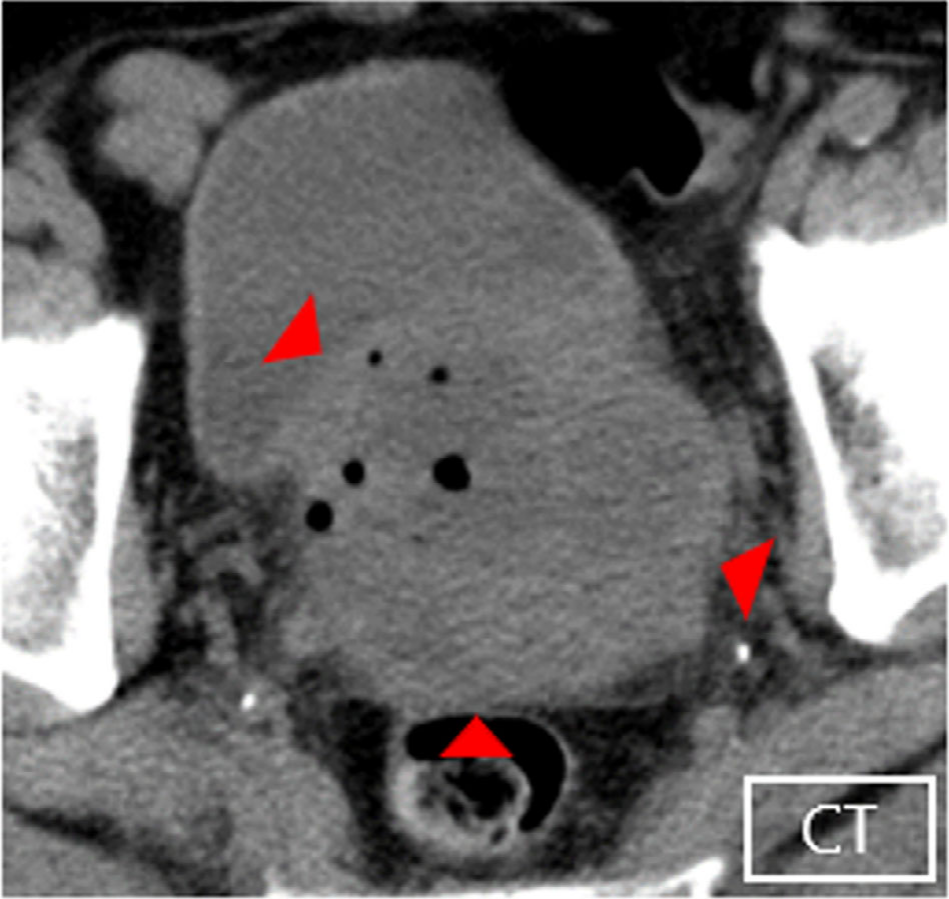
CT revealed a large multilocular mass with small amount of air.

**Fig. 3:**
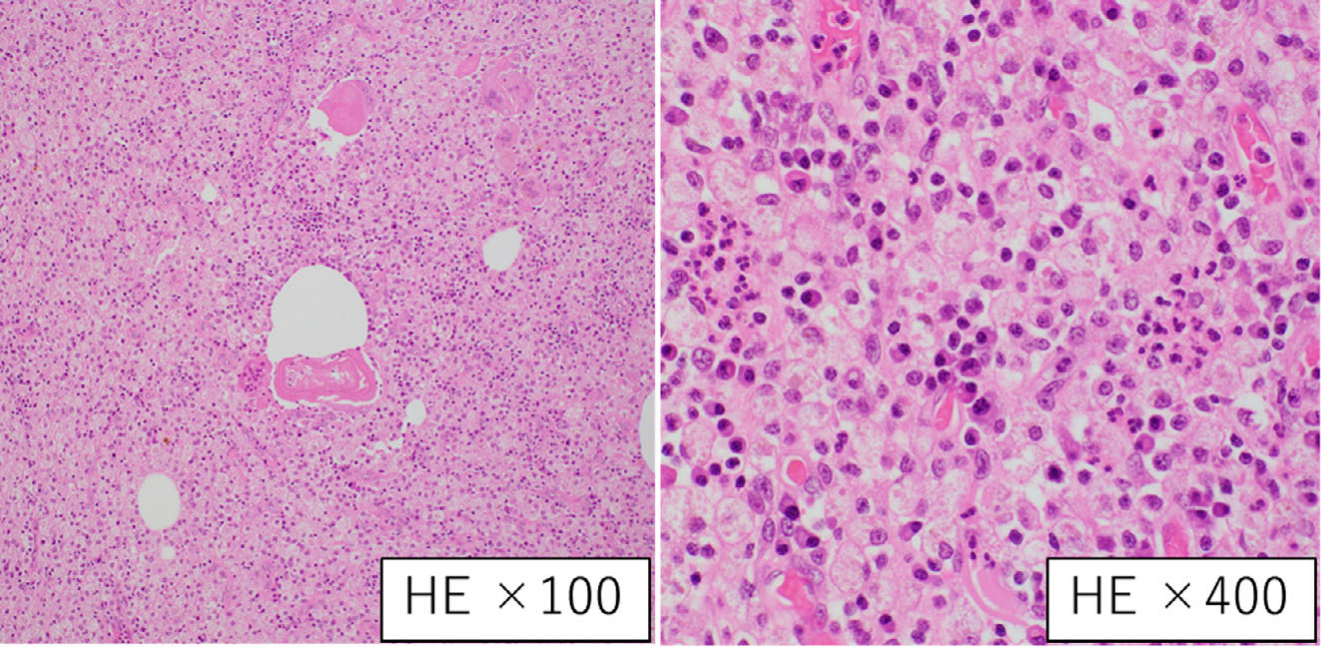
Microscopic findings of the tumor. Photomicrograph showing intense infiltration of prostate with lymphocyte, plasma cells, and xanthoma cells.

A 77‐year‐old man was admitted to our hospital because of a high prostate‐specific antigen level at his medical checkup. He had a history of diabetes mellitus and bladder cancer that was treated with transurethral resection of the bladder. He had no symptoms. His general physical examination was normal. Digital rectal examination indicated an enlarged hard consistency without nodules and tenderness. Prostate‐specific antigen level was 10.4 ng/mL (normal range: 0–4 ng/mL). Urinalysis date was normal. Ultrasonography revealed a large multilocular mass centered in the left of the prostate. MRI revealed a 6.5‐cm‐diameter multilocular mass with hemorrhage at the base of the left lobe of the prostate. There was an area with moderate T2‐weighted image signal and decreased apparent diffusion coefficient on the ventral side (Fig. 1). The PI‐RADS score was 3. Cystoscopy showed on obvious abnormalities. Transrectal ultrasonography‐guided systematic needle biopsy of the prostate was performed. There were 10 transperineal biopsies and four transrectal biopsyies. MRI‐fusion biopsy was not performed. Histopathological examination revealed no evidence of malignancy or XP. Five days after biopsy, the patient developed fever of 38°C. He had not subjective symptoms other than fever, such as dysuria or frequent urination. Blood test showed a prominent elevated inflammatory response. Urinalysus showed no pyuria. Enhanced CT revealed abscess formation in the prostate tumor. The patient was admitted to our hospital for treatment. Urine and blood cultures were negative. Transperineal puncture drainage was performed, but drainage was difficult. The patient was discharged with fever after antimicrobial administration due to inflammatory response improved a little. One month later, the patient developed fever again and was readmitted to our hospital. At this time, he had frequent urination. CT scan showed a gas image (Fig. 2), and we suspected recurrence of infection. Transperineal puncture drainage was performed, but drainage was difficult only in small amounts. The pus culture was *Escherichia coli*. To control infection, open suprapubic tumor resection was performed. Considering the size of the mass, we decided that urethral surgery would be difficult and decided on open surgery. Histopathology examination revealed xanthogranulomatous inflammation that was composed of foam cells, lymphocytes, and plasma cells and no evidence of malignancy, bacteria, fungi of special stainings such as Ziehi–Neelsen Dyeing (Fig. 3). Histopathological examination confirmed the patient's diagnosis of XP. At 15 months of follow‐up, he remains symptom‐free and CT revealed no abnormal findings.

## Discussion

XP is a very rare benign inflammatory lesion of the prostate that can be similar to prostatic carcinoma in clinical presentation and radiological characteristics. Since the first report of XP, less than 30 cases of XP have been reported in the English literature. Cases of prostate abscesses occurring at the same time as XP are even rarer, with only about five cases documented in the literature.^1^ This case was particularly unusual in that it showed a multilocular mass image on MRI, which are novel findings of the present study to the best of our knowledge. Considering that histopathological examination of the prostate biopsy revealed no evidence of XP, we also believe that the prostate biopsy may have caused XP. To the best of our knowledge, there have been no such reports.

The exact etiology of XP is unclear. Hyperlipidemia, autoimmunity, and blockage of prostate ducts have been suggested as etiological factors. Among them blockage of prostate ducts appears to play a major role in its pathogenesis.^2^ In the present case, as intravesical Bacillus Calmette–Guérin (BCG) therapy was performed, frequent mechanical manipulation was performed. Furthermore, the patient had autoimmunity disorder. This condition may have caused the XP.

The average age at diagnosis is in the early 60s. Clinically, patients often have urinary tract obstruction or severe lower urinary tract infection.^3^ Imaging may not differentiate between XP and prostatic adenocarcinoma.^4^ In addition, XP may increase serum prostate‐specific antigen levels, which is usually transient.^3^ Therefore, the final diagnosis should be based on histopathological examination. The treatment of XP should be conservative in principle, so it is important that the diagnosis is confirmed at the time of biopsy in order to perform conservative treatment. However, if the conservative approach fails, surgical treatment is sometimes required in about 10% of cases, as in the present case.

## Conclusion

We experienced a case of XP with multilocular mass image on MRI.

## Author contributions

Takashi Okamoto: Writing – original draft. Kazuki Doi: Writing – review and editing. Soma Ogura: Writing – review and editing. Masafumi Kusunose: Writing – review and editing. Kosuke Takahashi: Writing – review and editing. Takuya Fujimoto: Writing – review and editing. Mototsugu Muramaki: Writing – review and editing. Yuji Yamada: Writing – review and editing.

## Conflict of interest

The authors declare no conflicts of interest.

## Approval of the research protocol by an Institutional Reviewer Board

This study was approved by the Institutional Review Board of Hyogo Prefectural Amagasaki General Medical Center and was conducted in accordance with the tenets of the Declaration of Helsinki. All specimens were collected from the patients after their written consent was obtained.

## Informed consent

Informed consent was obtained from the patient.

## Registry and the Registration No. of the study/trial

N/A.
